# Editorial: Advances in the endocrine role of the skeleton volume II

**DOI:** 10.3389/fendo.2023.1345813

**Published:** 2023-12-20

**Authors:** Michela Rossi, Helen J. Knowles, Andrea Del Fattore

**Affiliations:** ^1^ Bone Physiopathology Research Unit, Translational Pediatrics and Clinical Genetics Research Division, Bambino Gesù Children’s Hospital, IRCCS, Rome, Italy; ^2^ Botnar Institute for Musculoskeletal Sciences, Nuffield Department of Orthopaedics Rheumatology & Musculoskeletal Sciences, University of Oxford, Oxford, United Kingdom

**Keywords:** bone, bone disease, endocrinology, osteoclast, osteoblast

In the last years, the identification of crosstalk between bone tissue and other organs and the analysis of its involvement in different diseases, have been of great interest to the research community. Indeed, from the archaic description of the skeleton exclusively as a support and protective scaffold for the body, this research has discovered novel functions associated with bone and has dissected the mechanisms of its interplay with other organs (1, 2).

In the paper published in this Research Topic, Cui et al. investigated the causal association between non-alcoholic fatty liver disease (NAFLD) and osteoporosis. NAFLD comprises of steatosis, non-alcoholic steatohepatitis, and fibrosis in obese children and adolescents. Interestingly, the possible correlation between NAFLD and osteoporosis has already been explored by previously published papers with inconsistent results (3). In this paper, the authors employed a Mendelian randomization analysis to evaluate the potential causal effect of NAFLD on the development of osteoporosis, fractures, and falling risk, and discovered that the genetic prediction of NAFLD was linked with an increased risk of osteoporosis without observing a correlation between NAFLD variants and fractures and falling risk.

Moreover, Xiong et al. investigated the link between thyroid dysfunction and bone tissue, analysing a potential correlation between thyroid dysfunction and hallux valgus (HV, bunion). Although thyroid diseases have widespread systemic manifestations, including the alteration of bone metabolism (4), the causal effect of thyroid alterations on hallux valgus is still unknown. The etiology of HV is currently under investigation since many factors can contribute to the development of this forefoot deformity including genetic factors, improper habits, inflammatory and neuromuscular joint disease. The authors performed a Mendelian randomization analysis considering hypothyroidism, hyperthyroidism, thyroxine (FT4; free T4) and thyroid stimulating hormone (TSH) as exposure and HV as outcome. They found that high risk of Hallux valgus recurrence is observed in hypothyroidism, without revealing correlation with hyperthyroidism, FT4 and TSH.

Also in this Research Topic, Wang et al. investigated the interaction between obesity and diabetes with bone alterations. Using data from the National Health and Nutrition Examination Survey (NHANES), the authors revealed a potential correlation between weight-adjusted waist index (WWI) and total BMD (Bone Mineral Density) in nearly 7,000 US adolescents. They performed a multivariate linear regression analysis that revealed a negative correlation between WWI and total BMD; they also detected an L-shaped association between WWI and total BMD, with a flex point at 9.98 cm/√kg.

Additionally, Li et al. analysed how type 1 diabetes could influence periodontal ligament and alveolar bone remodeling during axial tooth movement in mice. Using streptozotocin (STZ)-injected mice, the authors investigated how axial tooth movement was inhibited in type 1 diabetic mice, potentially due to alterations of the periodontal ligament collagen fibers or to osteoclasts.

The endocrine role of osteocalcin was also investigated within this Research Topic. Verdelli et al. analysed how osteocalcin regulates the function of parathyroid tumor cells. They performed *in vitro* experiments with primary cells isolated from parathyroid adenomas and HEK cells, and reported how both osteocalcin with gamma-carboxyglutamic acid residues (GlaOC) and the uncarboxylated form (GluOC) control pERK/ERK and active β-catenin, mainly through the activation of the calcium-sensing receptor (CASR).

Bone remodelling can be influenced by external factors including lifestyle choices such as inadequate sun exposure, minimal physical activity, malnutrition, smoking, or excessive alcohol intake, as well as environmental factors that can adversely affect bone health and contribute to bone loss (5). In the elegant review published by Romano et al., osteoporosis and dermatoporosis are described as consequences of the aging process respectively in the bone and skin and share vitamin D deficiency. The term “Dermatoporosis” was introduced about 15 years ago to describe a condition characterized by thinner skin that becomes fragile and tends to rip, leading to deep dissecting hematomas (6). Vitamin D has well-established effects on bone tissue but further investigation is required to understand how it regulates aging in other tissues, including the skin, to decipher the role of Vitamin D deficiency in the development of dermatoporosis.

Moreover, in the paper published by Zhang et al., a cross-sectional study on a Chinese cohort of 3433 individuals was performed to evaluate the effects of cooking oil fumes (COFs) and fume extractors on BMD. They found significant correlations between the non-use of fume extractors and total Lombar Spine BMD, as well as bone formation markers including PINP (Procollagen type I N-terminal propeptide) and ALP(Alkaline phosphatase).

In this Research Topic, advances in the understanding of bone physiology were also reported, directing attention to the potential translational impact of bone endocrinology. Janssen et al. investigated gene expression in calvarial and cortical bone of juvenile female mice. They did not report alterations in classic bone gene expression (e.g. Runx2, Osx, ALP, Col1a1, Col1a2 and Dentin matrix protein 1) between calvaria and cortical bones. However, they did find alterations in genes involved in skeletal diseases, craniosynostosis and weight loading.

Currently, research on ferroptosis may hold promise for the prevention and treatment of primary osteoporosis. Ferroptosis is an iron-dependent cell death and it differs from apoptosis, necrosis, autophagy, and other forms of cell death as it is caused by unrestricted lipid peroxidation and accumulation of reactive oxygen species (ROS). Using bioinformatic methods, Xia et al. identified 5 key ferroptosis-DEGs (differentially expressed genes) associated with primary osteoporosis and ferroptosis, including SIRT1, HSPA5, MTOR, HIF1A and BECN1.

This Research Topic is of interest both to basic and clinical researchers since it takes into consideration a wide range of topics regarding bone research. Original studies and reviews have been published to describe the latest discoveries in skeleton physiology and function.

## Author contributions

MR: Writing – original draft. HJK: Writing – original draft. ADF: Writing – original draft, Writing – review & editing.
